# Paranoia, with Delusions of Change in Sex1Read before the American Medico-Psychological Association, Chicago, June, 1893.

**Published:** 1893-11

**Authors:** C. B. Burr

**Affiliations:** Medical Superintendent of the Eastern Michigan Asylum


### Buffalo Medical and Surgical Journal

Vol. XXXIII. NOVEMBER, 1893. No. 4.

### PARANOIA, WITH DELUSIONS OF CHANGE IN SEX.[1]

### PSYCHOLOGICAL OBSERVATIONS AND SURGICAL NOTES.

By C. B. BURR, M. D.,
Medical Superintendent of the Eastern Michigan Asylum.

I believe that alienists generally will bear me out in the statement, that delusions of sexual change are of relatively rare occurrence. In an experience with the insane in a large State asylum, extending over a period of many years, I can call to mind but two or three cases presenting these symptoms. In view of this, the study of the genesis of such delusions comes to my mind as a matter of considerable interest.

A. P., a woman, single, aged 36 at the time of her admission, came under observation in the Eastern Michigan Asylum in 1885. She had always shown a lack of application and adaptability, was naturally suspicious and apt to misconstrue motives, was erratic, and had met with numerous failures and disappointments. Mental disease was said to have existed for ten years, and for the three years immediately preceding her admission rational intervals had been wanting. A period of great depression, succeeded by excitement and the development of extravagant delusions, occasioned her admission to the asylum. She was suspicious and thought her life threatened. She denied relationship with the members of her family, and called herself "Queen Anne." At one time she expressed the delusion that she had been ravished. She alleged conspiracy on the part of her relatives, claimed that she was an adopted daughter of R. P., and that he, dying, had made her sole legatee. Upon this she based her claim to all the property in possession of the family. "Some one" had told her, ten years ago, that there was a vault in the house filled with gold that belonged to her, and this she made unsuccessful attempts to find. She did not explain the reasons for believing herself of royal lineage, but alleged that if opportunity were offered she could prove it. She displayed a reluctance to comply with the rules of the Institution because of superiority to those about her and to all regulations, signed herself "Princess," was selfish, and would work for herself only. In July, 1886, about one year after her admission, physical failure was noticed. Her

1. Read before the American Medico-Psychological Association, Chicago, June, 1893.

countenance became sallow and she lost flesh, but opposed any investigation of her case. From this condition recovery soon took place. In August of the same year she expressed the delusion that she was the mother of ten children, became more elated, signed herself "Lady A——," disposed of hall property, on the ground that it was hers, by throwing it out of the windows, and was pleased to discard her night-dress after one night's wearing. She expressed delusions of poison. In the year 1887 she was more industrious, but was inclined to seclude herself, and closed the opening to the ventilating flue with a cloth to prevent the "hot gas" from descending. She called herself "Richard the Lion-Hearted's Daughter." Her physical health was fair, but on one occasion she had an attack of vomiting, the cause of which could not be discovered. In 1888, she developed the delusion that there was a child concealed in her mattress, and frequently tore it open under that impression. In the same year the note is made that she "is apparently losing mental force, and her conversation has less point and coherency." She still disclaimed relationship with her family, and was alternately patronizing and rude toward her relatives when they visited her. In September, 1888, physical failure is again noted. She complained of bloating and pain in the abdomen. Her pulse was weak and intermittent, and she suffered from menorrhagia and metrorrhagia, an interval of but two weeks occurring between periods of flowing. There was also present at this time a sub-acute rheumatism, referred to the knees. In the following January, 1889, she declared that her head was "filled with babies." On one occasion she contracted a cold, from exposure consequent upon removing her mattress from the bed, under the impression that there were "young ones in it that might crawl into" her. She asserted that she was the "fourteenth daughter of King James," that her name was "Iceldoor," because her father was born in the arctic regions as the result of union of the temperate and frigid zones. In March she had a sensation in the abdomen, which caused her to request the physician to listen to "hear the young ones." In April, 1891, there occurred a complete change in these impressions. She then, for the first time, claimed that she was a man, desired to be called "Mr. Leslie," and alluded to her clothing as if she thought it were that of a man. Later she wished to be known as "Lord Halton," showed a marked preference for the society of men, and was extremely indignant toward the attendants for bestowing upon her ordinary attentions. She sat in her room much of the time, assuming man-like attitudes, wore her dresses looped up above her shoe-tops, and parted her hair on one side; declined to walk with the party, because out of place with women, and demanded that a barber come upon the hall to shave her at regular intervals. On the day of the fire at the Asylum she separated herself from the party as it was passing from one hall, through the center building, to another. Her absence was discovered, and when asked what her purpose was, she replied that she was about to get a lunch for the firemen. At night she plead law. In March, 1892, she expressed the delusion that there were children in her body, which could not be born because children were only born of women. Her physical health became much improved, although the flow from the uterine cavity was profuse, frequent, and irregular. In April, while convalescing from a fever, she complained of pain in her right side. Examination showed an enlarged abdomen, over which there was an extended area of dullness, and two small tumors, apparently in the abdominal wall. Examined later by Dr. Manton, consulting gynecologist, the presence of a solid tumor, probably in connection with the uterus, was diagnosticated. During the months of May and June she suffered from suppuration of the middle ear. This was succeeded by a collection of pus posterior to the ear, and sub-periosteal, which required surgical interference. After recovering from the operation she improved much physically. During subsequent investigations of the abdomen she explained that at some previous time she had been operated upon and her genitals made to conform to those of a female. She spoke of her vagina as a wound. The abdominal enlargement increased very rapidly.

In March, 1893, laparatomy was performed by Dr. Manton. An incision four inches in length was necessary, because of the large size and irregular shape of the tumor. This proved to be a uterine fibro-cyst, weighing seven pounds. There was no pedicle in connection with the tumor, and, in extracting it, removal of much of the uterine tissue was necessary. A Koebele's serre-nœud was applied at the level of the internal os. After the removal of the tumor, the abdomen was thoroughly washed out with hot water, the stump fixed with pins fastened in the lower angle of the wound, and the abdominal incision closed. She has recovered rapidly, and without incident of any surgical importance, is now able to be about the hall, and is in every respect more comfortable than previous to the operation. For some time following it she objected to attentions from the nurses, and was extremely improper in her remarks to those who came near her, because of the impropriety of their presence in the sick-room of a man. She insisted upon being called "Jim Michilimackinac," and was extremely irritable if spoken of as "she" or "her," or called by her own name. Since getting up from bed, however, her mental improvement has been of the most marked and conspicuous character. She is less irritable toward those about her, is pleasant when addressed, and takes an interest in hospital matters. Previous to the operation she was irritable, inclined to seclude herself, unwilling to converse, and did not show interest in anything outside of her delusions. She now seeks the company of others, enters pleasantly into their conversation, and avoids no one. While still contending that she is a man, and asserting that many years ago an operation was made with the effect of unsexing her, her assertions are less irritable made, and arguments to show the falsity of her belief are not met, as heretofore, with anger, rage, and vituperation. Her avoidance, in the past, of the society of women, arose, doubtless, in a measure from her delusion that she was of the opposite sex. While this delusion still persists, it does not lead her to the same conduct. For the first time in the many years she has been under observation, she is on friendly terms with her attendants, seems to take delight in doing little things which they request, and shows an affectionate disposition. Previous to the operation she was restless at night and inclined to sit up in bed and talk. Now she sleeps soundly the night through. She no longer speaks of babies in her abdomen or in her mattress.

In view of the size and character of the tumor, and the liability to attacks of peritonitis which constantly menaced the patient, the removal of the tumor was undertaken none too soon, and the results of the operation, surgically speaking, have been highly gratifying. Whether the absence of the tumor will correct the delusions in respect to sex, it is yet too early to predict. Such manifest improvement in her general mental condition having already occurred, however, much is hoped for.

If, as Ribot contends, "the personality results from two fundamental factors—the bodily constitution, with its tendencies and feelings, and the memory," we must, I think, seek for the genesis of this delusion in the changed sensations arising from the sexual organs in consequence of its presence. Lallemant, quoted by Ribot, records the case of a patient who believed himself to be a woman, and who wrote letters to an imaginary lover. At the autopsy there was found hypertrophy with induration of the prostate, and alteration of the ejaculatory glands. Ribot makes the assertion that it is probable that in many cases of this kind there has been a perversion or abolition of the sexual feelings.

That the delusion as to the change in sex developed about the same time that the uterine fibroid began to make serious encroachments, seems reasonable to conclude from the history of the case, the delusion having been present for about two years. While metrorrhagia had occurred previous to that time, it is probable that the tumor had not before acquired a growth sufficient to determine any marked alteration in the organic sensations proceeding from the pelvic viscera.

